# Evaluation of shape, size, and location of mental foramen in dentulous and edentulous among Saudi population using 3D cone-beam computed tomography

**DOI:** 10.12688/f1000research.74434.1

**Published:** 2022-08-09

**Authors:** Mlook Ghazi AlOtaibi, Ahmad Tawfig, Hassan Mohamed Abouelkheir

**Affiliations:** 1 College of Dentistry, Riyadh Elm University, Riyadh, 13781, Saudi Arabia

**Keywords:** Mental foramen, CBCT, dentulous, edentulous

## Abstract

**Background**: Mental foramen (MF) and its accessories are the important anatomical considerations while placing implants or doing surgical procedures in and around the mandibular premolar region. This study aimed to evaluate the shape, size, and location of mental foramen in dentulous and edentulous patients among the Saudi population using 3D cone-beam computed tomography (CBCT).

**Methods**: In this retrospective study, CBCT scans that were taken between 2015 and 2020 from Riyadh Elm University were retrieved. A total of 180 samples of CBCT (90 dentate and 90 edentulous) were taken. Prevalence of different horizontal positions of the mental foramen (P1 to P6) and other additive parameters like the distance from mental foramen to alveolar crest and inferior border of the mandible, along with the mental foramen angle was assessed. The difference in the mental foramen location among dentate and edentulous subjects was assessed. Gender and age variation also was assessed. All the data were statistically analyzed using SPSS.

**Results:** The predominant horizontal position is P4 followed by P3 (59 % in males and 63 % in females at P4, and 15 % each in males and females at P3 respectively). The horizontal position of the mental foramen and gender showed a statistical significance difference, especially at the P3B, P5, and P4 positions. Moreover, a statistically significant difference was seen in the mental foramen to the mandibular inferior border of the mandible (MF_MSB) and the width of mental formane in the transverse section (MFW). Comparison of the mental foramen among dentate and edentulous subjects showed a statistically significant difference. There was a change in the mental foramen with age.

**Conclusion:** Based on the methodology and sample of this study, it can be concluded that the edentulism only reduced the dimension of the mental foramen opening.

## Introduction

From our experience, the demand for seeking dental implants has grown so much that there has been a substantial increase in the surgical procedures being performed in the mandible. This requires the dentist to visualize the anatomic structure with precision to avoid any complications. As the popularity of implants increases, more challenges and complexity will need to be accepted by the implantologist and oral and periodontal surgeons. Modern imaging techniques are available, with their maximum utilization to assess and explore in-depth these difficult areas. ^1^

Taking precautions before attempting the surgical procedures by clarifying and categorizing the anatomy appropriately will prepare the clinicians better to handle any complications which may arise during and after the procedure as the mandible and maxilla house many anatomical structures. The posterior maxilla often presents issues with a poor quality of bone. In addition, pneumatization of the maxillary sinus further complicates the implant placement, and restoring this area becomes a difficult task. In the anterior maxilla, a lack of facial bone creates esthetic concern, and it is difficult to achieve an emergence profile. If the nasopalatine canal is sufficiently large, it may pose difficulties in placing the implant in this region, with potential complications which may arise later. ^2^

Compared to the maxilla, the mandible is further complicated. The posterior mandible poses a unique challenge for many surgical procedures and implant placements, because of the location of many important anatomical structures which need to be kept in mind. Most examples of the posterior region of the mandible present with an external oblique ridge, lingual concavity, and close association of lingual nerve in the third and second molar region, making the surgical procedure relatively cumbersome. Failure to detect the lingual concavity may force the implant placement outside the border of the mandible with possible fracture of the mandibular margin. ^3^

Besides, negligence in not considering the anatomical landmarks that are most closely associated with dental implant surgery like the mandibular foramen, the inferior alveolar nerve, the mental foramina, the mental nerve, the lingual nerve, and the incisive canal and its associated neurovascular bundle may end up with the patient exposed to possible nerve injury and complications associated with the neurovascular bundles, including hemorrhage. ^4^

The inferior alveolar nerve and artery in the mandibular canal begins at the mandible foramen and divides into two branches. The mental foramen is typically located on the lingual side between the premolars and the bottom border of the jaw. ^3^ The portion of the inferior alveolar nerve which presents anteriorly to the mental foramen, before exiting the canal, is referred to as the anterior loop of the inferior alveolar nerve. ^5^

The anterior canal and the submandibular fossa convexity are important anatomic factors that can limit useful bone in the posterior region, necessitating surgical augmentation or other critical surgical treatments. Instrumentation through the lingual cortical plate which is inadvertent or careless can bring about vascular harm that can cause the formation of a sublingual or submandibular hematoma, excessive bleeding, or infection. ^6^ In addition, information about the location of the mental foramen can lead to more accurate local anesthesia techniques and can avoid damage to the mental nerve during implant placement. ^3^

The lower jaw remodels throughout life with growth with recognized modifications in prominent places such as the mental foramen, mandibular foramen, and mandibular canal. The shifting of the position of the mental foramen with age is a well-established fact that has been confirmed by the majority of researchers on the subject. ^7^ Further, after the extraction and subsequent resorption of the local alveolar ridge, the mental foramen location may change move closer to the alveolar bone ridge and is thus more prone to damage during surgical procedures. ^8^ ^,^ ^9^ However, the typical location of the mental foramen is reported to be either between the apices of the first and second premolars or below the apex of the second premolar. ^3^ This position of the mental foramen demonstrates anatomical variations, which can be found as far anterior as the canine ^10^ and as far posterior as the first molar. ^11^

The mental foramen is an important anatomical structure representing the termination of the mandibular canal. ^12^ Its accurate identification depends on knowledge of its location; this is strategically important for diagnostic and clinical procedures. ^13^ As a result, it appears that no single and consistent pattern of mental foramen placement exists across diverse groups. Hence, in clinical dental treatment, a detailed assessment of the mental location typical of each group is extremely useful. The shape, size, and position of the mental foramen must be evaluated, especially when considering various dental treatments performed in the mandible.

Currently, high resolution 3D cone-beam computed tomography (CBCT) is the most promising and accurate technique available for quantitatively determining the location of mental foramen and the presence of anterior loops. ^14^

The high image quality of bone tissue and anatomical structure features provided by CBCT analysis reduces the risk of lesions in the lower alveolar vascular nerve bundle. Paralysis and bleeding may result in the front area of the mandible and nearby tissues. ^15^

Many authors and researchers in their retrospective studies assessed the details of the mental foramen (MF). ^16^ ^–^ ^19^ They have found that in very few cases, the exact position of the mental foramen can be observed in coronal, axial, sagittal, cross-sectional, and three-dimensional reconstructed images using CBCT.

Moreover, the CBCT easily overpowers drawbacks of panoramic radiograph like inaccurate patient placement due to a lack of standardization in placement throughout radiography, fluctuation in anatomic structures, and situations such as interruption and magnification in the radiographs, as well as some discrepancies in measuring the length.

It has been reported in the literature that race and ethnic variation are also visible in the mental foramen, accessory mental foramen, and anterior loop. ^17^ Considering this, there are variations expected in the Saudi population, yet few studies have reported variations. ^18^ ^,^ ^19^ There are reported studies using panoramic radiographs, however, such studies may not be useful in the digital era with 3D imaging providing clearer details in comparison. Thus, this retrospective study was conducted to evaluate the shape, size, and location of mental foramen in dentulous and edentulous among the Saudi population using 3D CBCT. This study aimed to compare the positions and dimensions of MF openings between edentulous and dentate subjects matched by gender and nationality through CBCT.

## Methods

### Ethical approval

This study was approved by the Ethics Committee of the College of Dentistry (IRB number FPGRP/2020/508/318/319), Riyadh Elm University, Riyadh, Kingdom of Saudi Arabia. All patients sign a consent form before their appointments which states that radiographs and photographs are property of the college and may be used for teaching clinical demonstrations or scientific publications.

### Sample collection

In this retrospective study, after due permission was obtained from the medical superintendent of the hospital, CBCT data was accessed. CBCT scans were taken between 2015 and 2020 from Riyadh Elm University (Database in Riyadh, Kingdom of Saudi Arabia). After receiving permission from the institution. Patient MR (Medical Record) numbers were used to trace the scans. The required sample size was calculated using G-POWER 3.1.9.7 program based on the findings from a previous study. ^20^ Cohen’s d effect size by dentulous and edentulous jaw difference in males and females was 0.70 and 0.90, respectively. A minimum required sample of (n=180) was considered. At first, we procured around 500 CBCT with our area of interest. Each CBCT was given a number from 1 to 500. CBCT could be traced back if required based on the hospital number (MR number). A total of 500 scans were assessed and 180 scans fulfilling the inclusion criteria were selected. The following inclusion criteria were used to select the final 180 samples of CBCT from the source data:

▪ Only Saudi nationals
▪ Free from any systemic condition or systemic conditions which may have an impact on the result of the study
▪ Only permanent dentition
▪ Good quality CBCT with high volumetric data

Once 180 samples were selected, the samples from these criteria were then evenly sorted into two categories; group 1: dentate subjects; and group 2: edentulous subjects (unilateral or bilateral missing premolars). Variables such as gender and age were also considered in the group categories.

After screening and consideration of inclusion criteria, a total of 180 CBCT were included. In both groups the following measurements were taken following a previous study. ^20^ No new measurements were taken in the course of this study:

*CBCT data*: Images were taken with a Sirona Galileos CBCT machine with exposure setting (85 kV, 28-35 mAs). CBCT images were exported in a digital imaging communication in medicine (DICOM) file format (.dcm). Images were accessed through on-demand 3D reconstruction imaging software (Version 8.0 205686 CyberMed inc, Seoul, Korea). Using CBCT panoramic reformatting images, tangential as well as cross-sectional view and nerve marking was done.

*Radiographic evaluation:* All the radiographic measurements were done by a single experienced CBCT reading examiner based at the university who is well trained in CBCT. They recorded the relevant measurements outlined below. To measure the reliability of the taken measurements, a second experienced examiner was asked to take the same readings following the described outlines for 20% of the final CBCT image scans. Inter-examiner reliability was assessed using Cronbach alpha before starting the assessment and considered acceptable (α>70%).

The following measurements were taken using the modified method proposed by Zaman *et al.* (2016). ^21^

i. The vertical position of the mental foramen
  Three vertical lines were drawn on the longitudinal axis of the first premolar, second premolar, and mesial root of the first molar, respectively, to identify the horizontal position of the mental foramen. A horizontal line was drawn connecting the apices of the first and second premolars.
  The vertical position of MF was measured as follows:
  • Level A (LA) - Above the horizontal line.
  • Level B (LB) - At the horizontal line.
  • Level C (LC) - Below the horizontal line.
  • Mental foramen - Alveolar crest in mm.
  • Mental foramen - Apical point of lower cortical mandibular bone is also measured.
ii. The horizontal position of the mental foramen
  • Position 1 (P1) - Situated mesial to the long axis of the first premolar.
  • Position 2 (P2) - Situated in line with the long axis of the first premolar.
  • Position 3A (P3A) - Mesial 1/3 of position between the long axis line of the first and second premolar.
  • Position 3 (P3) - Middle 1/3 of position between the long axis line of the first and second premolar.
  • Position 3B (P3B) - Distal 1/3 of position between the long axis line of the first and second premolar.
  • Position 4 (P4) - Situated in line with the long axis of the second premolar.
  • Position 5 (P5) - Between the long axis of the second premolar and mesial root of the first molar.
  • Position 6 (P6) - Situated in line with the long axis of the mesial root of the first molar.
iii. Additional parameters
  These are called additional parameters as they will adjunct the readings of primary parameters.
  • MF-MIB in mm (mental foramen to mandibular inferior border of mandible).
  • MF-MSB in mm (mental foramen to alveolar crest).
  • MFW (width) width of mental foramen in transverse section.
  • Emerging MF angle.

### Data analysis

Descriptive statistics for frequency, percentages, mean, and standard deviation were calculated for the various measures related to the mental foramen. For independent samples, a t-test was applied to compare mental foramen size, angle, and width between dentulous and edentulous jaws. The Mann Whitney U test was applied to compare the mental foramen- related continuous variables on right and left sides, and between gender. All the data were analyzed by using SPSS version 25 (IBM-SPSS, Armonk, NY: USA, RRID:SCR_016479). A p-value of <0.05 was considered statistically significant.

## Results

**Figure 1.**
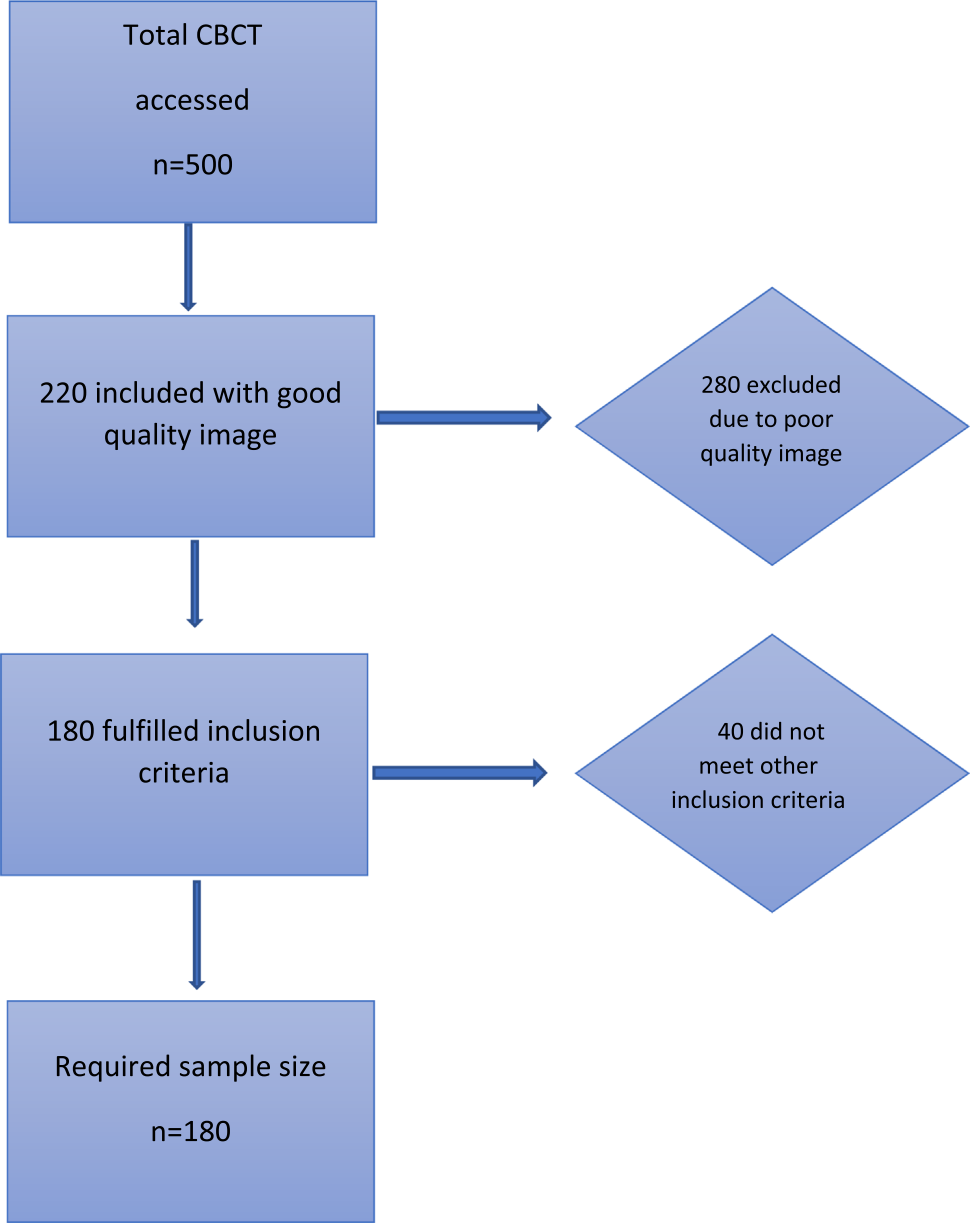
Participant selection. CBCT=cone-beam computed tomography.

From the total 500 scans, 280 were excluded as they were not good quality images and 40 as they did not fulfill other inclusion criteria such as Saudi nationals (n=22), free from systemic disease/condition (n=12), and permanent dentition (n=6). Hence, 180 were included in the analysis ( Figure 1).

The descriptive characteristics of the study variables are shown in Table 1. A total of 180 CBCT were analyzed, of which 96 were male and 84 were female. ^34^ Among 180 CBCT, it was ensured that there was equal distribution between dentate, edentulous, and sides (left and right) and vertical position among the CBCT is LC, horizontal position shown P4 having the highest percentage 67.8%.

**Table 1.** Characteristics of the study variables.

Variables		n	%
Gender	Male	96	53.3%
	Female	84	46.7%
	Total	180	100.0%
Group	Dentate	90	50.0%
	Edentulous	90	50.0%
	Total	180	100.0%
Side	Left side	180	50.0%
	Right side	180	50.0%
	Total	360	100.0%
Vertical Position	LC	180	100.0%
	Total	180	100.0%
Horizontal Position	P1	0	0.0%
	P2	4	1.1%
	P3A	2	0.6%
	P3	60	16.7%
	P3B	14	3.9%
	P4	244	67.8%
	P5	34	9.4%
	P6	2	0.6%
	Total	360	100.0%

• Level C (LC) - Below the horizontal line. • Position 1 (P1) - Situated mesial to the long axis of the first premolar. • Position 2 (P2) - Situated in line with the long axis of the first premolar. • Position 3A (P3A) - Mesial 1/3 of position between the long axis line of the first and second premolar. • Position 3 (P3) - Middle 1/3 of position between the long axis line of the first and second premolar. • Position 3B (P3B) - Distal 1/3 of position between the long axis line of the first and second premolar. • Position 4 (P4) - Situated in line with the long axis of the second premolar. • Position 5 (P5) - Between the long axis of the second premolar and mesial root of the first molar. • Position 6 (P6) - Situated in line with the long axis of the mesial root of the first molar.

59 males and 63 females showed P4 horizontal position. A statistically significant association was found between the horizontal position of the mental foramen and gender (p<0.05) ( Table 2). A statistically significant association was also found between the horizontal position of the mental foramen and dentate status (p<0.05) ( Table 3). On the other hand, no statistically significant association was found between the horizontal position of the mental foramen and sides (p>0.05) ( Table 4).

**Table 2.** The horizontal position of the mental foramen between gender.

		P2	P3A	P3	P3B	P4	P5	P6	Total	p value
Male	n	2	0	15	7	59	12	1	96	0.016
	%	1.1	0.0	8.3	3.9	32.8	6.7	0.6	53.3	
Female	n	0	1	15	0	63	5	0	84	
	%	0.0	0.6	8.3	0.0	35.0	2.8	0.0	46.7	
Total	n	2	1	30	7	122	17	1	180	
	%	1.1	0.6	16.7	3.9	67.8	9.4	0.6	100	

**Table 3.** The horizontal position of the mental foramen between dentate and edentulous jaws.

		P2	P3A	P3	P3B	P4	P5	P6	Total	p value
Dentate	N	2	1	24	7	43	13	0	90	<0.001
	%	1.1	0.6	13.3	3.9	23.9	7.2	0.0	50.0	
Edentulous	N	0	0	6	0	79	4	1	90	
	%	0.0	0.0	3.3	0.0	43.9	2.2	0.6	50.0	
Total	n	2	1	30	7	122	17	1	180	
	%	1.1%	0.6	16.7	3.9	67.8	9.4	0.6	100.0	

**Table 4.** The horizontal position of the mental foramen between right and left sides.

		P2	P3A	P3	P3B	P4	P5	P6	Total	p value
Left side	n	4	2	22	8	126	16	2	180	0.378
	%	1.1	0.6	6.1	2.2	35.0	4.4	0.6	50.0	
Right side	n	0	0	38	6	118	18	0	180	
	%	0.0	0.0	10.6	1.7	32.8	5.0	0.0	50.0	
Total	n	4	2	60	14	244	34	2	360	
	%	1.1%	0.6	16.7	3.9	67.8	9.4	0.6	100.0	

The mean and standard deviation (SD) mental foramen angle was highest on the left side in the 50-59-year-old group in the edentulous group (53.95±13.97) and least on the right side in the 16-19-year-old group in the dentulous group (38.00±8.26) ( Table 5). The mean distance of the mental foramen to the alveolar crest (MF_MSB) was statistically significantly higher (t=6.235, p<0.001) in males (14.52±3.29 mm) than females (11.22±3.80 mm). In addition, the mean distance of the mental foramen to the mandibular inferior border of the mandible (MF_MIB) was statistically significantly higher (t=2.875, p=0.005) in males (11.59±2.10 mm) than females (10.25±3.95 mm). Moreover, the mean width of the mental foramen in the transverse section (MFW) was statistically significantly higher (t=3.622, p<0.001) in males (3.43±0.82 mm) than females (2.99±0.83 mm). However, the mean M angle between males (44.21±9.06) and females (46.11±12.03) was not found to be statistically significant (t=-1.205, p=0.239). The mean MF_MSB, MF_MIB, and MFW were statistically significantly higher in males (p<0.05) ( Table 6).

**Table 5.** The right and left mental foramen angle according to age.

Age (Years)	Side	n	Mean	SD
16-19	Left	6	43.90	8.66
	Right	6	38.00	8.26
20-29	Left	72	42.30	9.00
	Right	72	42.29	8.83
30-39	Left	30	43.61	12.30
	Right	30	41.09	8.16
40-49	Left	10	50.48	11.44
	Right	10	46.76	10.26
50-59	Left	26	53.95	13.97
	Right	26	52.02	11.43
60-69	Left	18	46.46	11.32
	Right	18	44.46	10.49
70-79	Left	18	50.78	10.57
	Right	18	46.24	6.20

**Table 6.** Comparison of study variables between gender.

		n	Mean	SD	SEM	t	p value
MF_MSB	Male	96	14.52	3.29	0.34	6.235	<0.001
	Female	84	11.22	3.80	0.41		
MF_MIB	Male	96	11.59	2.10	0.21	2.875	0.005
	Female	84	10.25	3.95	0.43		
MFW	Male	96	3.43	0.82	0.08	3.622	<0.001
	Female	84	2.99	0.83	0.09		
M angle	Male	96	44.21	9.06	0.93	-1.205	0.239
	Female	84	46.11	12.03	1.31		

MF-MSB (mental foramen to alveolar crest); MF-MIB (mental foramen to mandibular inferior border of mandible); MFW (width of mental foramen in transverse section); MF angle (mental foramen angle).

The mean distance of MF_MSB was found to be statistically significant (t=8.763, p<0.001) higher in dentate (15.11±2.72 mm) than edentulous (10.85±3.73 mm). On the other hand, the mean distance of MF_MIB between dentate (11.36±2.16 mm) and edentulous (10.57±3.90 mm) was not found to be statistically significant (t=1.688, p=0.093). The mean MFW between dentate (3.46±0.74 mm) and edentulous (2.99±0.89 mm) was found to be statistically significantly higher in dentate (t=3.815, p<0.001). Furthermore, the mean M angle between dentate (41.92±8.33) and edentulous (48.27±11.61) was found to be statistically significant (t=-4.217, p<0.001) ( Table 7). There was no statistically significant difference in the mean of all study variables by sides (p>0.05) ( Table 8).

**Table 7.** Comparison of study variables between dental status.

		n	Mean	SD	SEM	t	p value
MF_MSB	Dentate	90	15.11	2.72	0.29	8.763	<0.001
	Edentulous	90	10.85	3.73	0.39		
MF_MIB	Dentate	90	11.36	2.16	0.23	1.688	0.093
	Edentulous	90	10.57	3.90	0.41		
MFW	Dentate	90	3.46	0.74	0.08	3.815	<0.001
	Edentulous	90	2.99	0.89	0.09		
MF angle	Dentate	90	41.92	8.33	0.88	-4.217	<0.001
	Edentulous	90	48.27	11.61	1.22		

MF-MSB (mental foramen to alveolar crest); MF-MIB (mental foramen to mandibular inferior border of mandible); MFW (width of mental foramen in transverse section); MF angle (mental foramen angle).

**Table 8.** Comparison of study variables between sides.

		n	Mean	SD	SEM	t	p value
MF_MSB	Left	180	13.02	4.10	13.02	0.123	0.902
	Right	180	12.95	3.70	12.95		
MF_MIB	Left	180	10.61	2.15	0.23	-1.506	0.134
	Right	180	11.32	3.91	0.41		
MFW	Left	180	3.24	0.90	0.09	0.317	0.752
	Right	180	3.20	0.80	0.08		
M angle	Left	180	45.97	11.42	1.20	1.119	0.264
	Right	180	44.21	9.62	1.01		

MF-MSB (mental foramen to alveolar crest); MF-MIB (mental foramen to mandibular inferior border of mandible); MFW (width of mental foramen in transverse section); MF angle (mental foramen angle).

## Discussion

A detailed understanding of the anatomical structures of the mandible and maxilla are important during periodontal and implant surgical procedure. A good surgeon always plans the surgical procedure using required diagnostic aids and gives attention to each small anatomical structure in the surgical field, including anatomical variation (if any) to avoid surgical complications. ^3^ The mandible, with its associated anatomical structures in the posterior region, always requires additional attention to avoid injury to the neurovascular bundles and any complications related to them. The mental foramen and associated alterations should have been given attention before surgery. Anatomically, mental foramen shows a lot of variation with the number of foramina and accessory foramina and the possibility of the presence of an anterior loop of the mental nerve. ^3^

In the present study, we have utilized CBCT to assess the position of the mental foramen. Many previous studies evaluating the mental foramen and its associated structure were done in cadavers, and human studies using panoramic radiographs, ultrasonography, and CBCT. ^13^ ^–^ ^19^ Panoramic radiographs have their disadvantages with a lack of clarity, thus smaller mental foramen may go unnoticed. In the present study, When compared to multi-detector computed tomography (MDCT) imaging, CBCT was the radiographic method of choice for visualizing the hard tissues of the mandible because it reveals anatomical structures without superimpositions and deformation, which are observed in traditional imaging techniques such as panoramic image analysis and at low levels of radiation. ^22^

Although several studies are available regarding the mental foramen and its associated anatomical variation which are carried out within the Saudi population, there is still scope for more information to gather and add to the existing literature. ^16^ ^–^ ^19^ In addition, all the earlier studies done in Saudi Arabia were only among dentate subjects. This is the first study exclusively comparing the dentate and edentulous. Furthermore, initial studies were done using panoramic views. ^16^ ^–^ ^19^ However, there are drawbacks of using a panoramic view as previously outlined and the result may vary when they are compared with that of the CBCT. ^10^

The prevalence of horizontal position of mental foramen in the present study predominated by P4 (35% in males and 32.8% in females), followed by P3 (8.3% each in males and females). P4 appears to be the most common position, or more prevalent position, seen in almost all the studies which are done among Saudi, Jordanian, and Egyptian population. ^18^ ^,^ ^23^ However, variation in the position was seen in studies among the Indian (P5), European (P3), and Iranian population (P3). In the present study, the prevalence was similar to the study of Alam *et al.* (2018). ^18^ Many other studies have shown higher prevalence. The similarity and difference in the results are related to race and potentially due to change in the sample size of the age group. It may be possible that the sample size chosen is also responsible for the percentage of prevalence seen in different studies. The sample size of our study is less than the few studies mentioned above and more in a few other studies. Hence, the percentage of prevalence seen in this study may be different than the previous studies.

In the present study, the variation in the mental foramen compared between males and females was found to be statistically significant. The horizontal position of the mental foramen and gender showed a statistical significance difference, especially at the P3B, P5, and P4 positions. There was a statistically significant difference seen in MF_MSB and MFW. However, there is no significant difference in MF_MIB and MF angles. Differences in the position of mental foramen across gender and racial groups have also been reported in the literature. Present study results are similar to some parameters which were presented in Alam *et al.* (2018). ^18^ In their study, the P4 position is more prevalent in males which is similar to our study, but they have found P3 to be more prevalent in females. However, in our study, we have found P4 more commonly prevalent, followed by P3. There was a also statistical difference in P3B and P5 prevalence among males and females. A study done in the Indian population has shown slightly different results, where they have found P4 followed by P3 being common in males on the right side of the mandible and P4 for the females on the left side of the mandible. ^24^

Li *et al.* (2018) and Al-Mahalawy *et al.* (2017) found similar results to the present study with the difference in the prevalence among gender. ^17^ ^,^ ^25^ In a study of the Polish population, for the right or left side in males, the average values of a horizontal and vertical diameter were significantly higher on the right side than in the female subgroup. Whereas, on the left side the average value of only the vertical diameter was significantly higher in males compared to women. ^26^ Thus, various studies have pointed out the different or similar prevalences among males and females. Variability in the prevalence among males and females was not consistent, with few studies mentioning no difference and few other studies mentioning differences between the gender. The difference in the result could be related to the age group of the sample, sample size, and racial predilection.

The present study is unique compared to many other studies because we have taken both the dentate and edentulous patients. It is known that there is variation in the location of the mental foramen as the age advances due to resorption of the crestal bone loss or change in the position of the mandible due to differences in the growth of the mandible. In the present study, although we have found the prevalent position is P4 in both the dentate and edentulous groups, there was a statistically significant difference between these two groups. With regards to the age effect on the morphology of the foramina, studies have shown different results, although many studies did not specify the group as dentate or edentulous, the age group variation has been shown. ^16^ ^–^ ^19^ Differences in the division of the age group could lead to different conclusions ^27^ ^,^ ^28^ on variation in the mental foramen location, number of mental foramina, and anterior loop of the mental nerve.

One of the studies revealed that age-related differences of the accessory mental foramen size varied between pediatric and adult populations due to the growth process of the mandible ^29^; variation in the edentulous and dentate subject across age variation is an important consideration during implant placement. In the present study, there was no statistical significance in the MF position between the left and right sides in the horizontal position. However, we have seen the difference in the results for MF_ MSB, M angle, and MFW. The present study results are similar to the studies reported among the Jordanian, Saudi, and Iranian population, ^18^ ^,^ ^30^ but a few other studies reported the prevalence of asymmetric mental foramen in Saudi, Egyptian, and Jordanian populations too. ^10^ ^,^ ^20^ The variation in the position of mental foramen in different populations may be due to racial variations and factors which may have influenced the growth of either side in a different manner. ^18^

The variation in the distance between the mental foramen to the alveolar crest, relation to the lower border of the mandible, and corresponding changes in the width of mental foramen in the transverse section is commonly increased by age. The mean distance from the upper border of the mental foramen to the alveolar crest in our study is 15.11 mm in dentate and 10.85 mm in edentulous, which is almost in the range of the previous study done among the Saudi population ^17^ where the range is 9.1–19.2 mm (Mean: 14.3 mm). Similar reports have been published by Haktanir *et al.* (2010), which showed a mean distance of 14.2 mm (Range: 10.7–29.8 mm), ^31^ where the lower range is close to our study, but there was a much larger difference observed in the upper range.

Changes in the vertical distance seen in this study, especially in the upper range, could be due to the resorption of the alveolar crest, age of the patient, growth pattern changes in the mandible as the age advances, and genetic changes. ^17^ To overcome the shadow of the alveolar crest resorption having an impact on this parameter, one author suggested the use of cementoenamel junction (CEJ) of adjacent teeth as a guide. ^6^ Similar to the results of the relation between the mental foramen and alveolar crest, many studies established and presented the distance between the mental foramen and inferior border of the mandible. In our study, we have seen a mean distance of 10.57 mm in the edentulous and 11.36 mm in the dentate subjects. These study results are again similar to the study reports of Al-Mahalawy *et al.* (2017), where they reported the mean distance between the inferior margin of mental foramen and lower mandibular border was 13.8 mm (Range: 8.7–16.6 mm). ^17^ Similarly in the reports by Von Arx *et al.* (2013) and Kalender *et al.* (2012), the average distance was found to be 13.2 mm and 12.4 mm, respectively. ^32^ ^,^ ^33^

There is some limitation of the study. The sample size of the study though drawn after considering the value of previous studies, increasing the sample size may be helpful to draw a better conclusion. Further, we have considered dentate ane edentulous age group to a limited range, since the anatomy of mental foramen may vary from age to age. A further age range inclusion may broaden the result with a better conclusion. Considering the similarity in some of the parameters in the same ethnic population, differences across ethnic populations, changes in the age group, gender, and state of dentate and edentulous, a recent systematic review on the anterior loop and mental foramen rightly pointed that there is no fixed parameter to be relied on for the presence and distribution of anterior loop. ^13^ Though it appears that in a given ethnic population and age group some parameters appear to be similar, it is highly recommended not to rely on any average values available for the anterior loop. The clinician is advised to use imaging modalities available in every case wherever surgical procedure is to be performed near the mental foramen region for identification and accurate measurements of the anterior loop length to avoid any injury. ^13^ If clinicians want to avoid complications and extend comfort to the patient, it is prudent to be cautious during the operation. Clinicians should be on the lookout for unanticipated deviations, especially when doing dental operations that entail periosteal detachment and implant placement in the mental area.

## Conclusions

Within the limitation of the study, it can be concluded that the horizontal position of the mental foramen is predominated by P4 followed by P3 in this sample of the Saudi population. There is some variation in the mental foramen location across gender and different age groups, and also variation in the position of the mental foramen among dentate and edentulous subjects. Considering the variations in the age group, gender, and between dentate and edentulous subjects, the clinician needs to be careful about the position of the mental foramen and the use of CBCT as a diagnostic aid before the surgical procedure needs to be considered.

## Data availability

### Underlying data

Access to the underlying data is only available via the database in Riyadh Elm University. To access the database, researchers must contact the College of Dentistry in Riyadh Elm University to request access. Researchers must be the faculty of the said university in order to apply for data access to the authorities. A summary of the data used in this study is available in the underlying data statement.

Harvard Dataverse. Evaluation of Shape, Size and Location of a Mental Foramen in dentulous and edentulous among Saudi population Using 3D Cone-Beam Computed Tomography. https://doi.org/10.7910/DVN/LTOPGY. ^34^

This project contains the following underlying data:

• GR 1 dentate left.tab. (underlying data for group 1 dentate – left side)
• GR 1 R DENTATE.tab. (underlying data for group 1 dentate – right side)
• GR 2 LEFT.tab. (underlying data for group 2 endentulous – left side)
• GROUP 2 R.tab. (underlying data for group 2 endentulous – right side)

Data are available under the terms of the Creative Commons Zero “No rights reserved” data waiver (CC0 1.0 Public domain dedication).
