# Evaluation of shape, size, and location of mental foramen in dentulous and edentulous among Saudi population using 3D cone-beam computed tomography

**DOI:** 10.12688/f1000research.74434.2

**Published:** 2022-11-30

**Authors:** Mlook Ghazi AlOtaibi, Ahmad Tawfig, Hassan Mohamed Abouelkheir

**Affiliations:** 1 College of Dentistry, Riyadh Elm University, Riyadh, 13781, Saudi Arabia

**Keywords:** Mental foramen, CBCT, dentulous, edentulous

## Abstract

**Background**: Mental foramen (MF) and its accessories are the important anatomical considerations while placing implants or doing surgical procedures in and around the mandibular premolar region. This study aimed to evaluate the shape, size, and location of mental foramen in dentulous and edentulous patients among the Saudi population using 3D cone-beam computed tomography (CBCT).

**Methods**: In this retrospective study, CBCT scans that were taken between 2015 and 2020 from Riyadh Elm University were retrieved. A total of 180 samples of CBCT (90 dentate and 90 edentulous) were taken. Prevalence of different horizontal positions of the mental foramen (P1 to P6) and other additive parameters like the distance from mental foramen to alveolar crest and inferior border of the mandible, along with the mental foramen angle was assessed. The difference in the mental foramen location among dentate and edentulous subjects was assessed. Gender and age variation also was assessed. All the data were statistically analyzed using SPSS.

**Results:** The predominant horizontal position is P4 followed by P3 (59 % in males and 63 % in females at P4, and 15 % each in males and females at P3 respectively). The horizontal position of the mental foramen and gender showed a statistical significance difference, especially at the P3B, P5, and P4 positions. Moreover, a statistically significant difference was seen in the mental foramen to the mandibular inferior border of the mandible (MF_MSB) and the width of mental formane in the transverse section (MFW). Comparison of the mental foramen among dentate and edentulous subjects showed a statistically significant difference. There was a change in the mental foramen with age.

**Conclusion:** Based on the methodology and sample of this study, it can be concluded that the edentulism only reduced the dimension of the mental foramen opening.

## Introduction

From our experience, the demand for seeking dental implants has grown so much that there has been a substantial increase in the surgical procedures being performed on the mandible. This requires the dentist to visualize the anatomic structure with precision to avoid any complications.

The lower jaw remodels throughout life with growth which leads to recognized modifications in prominent places such as the mental foramen, mandibular foramen, and mandibular canal. The shifting of the position of the mental foramen with age is a well-established fact that has been confirmed by the majority of researchers on the subject. ^1^ Further, after the extraction and subsequent resorption of the local alveolar ridge, the mental foramen location may change and move closer to the alveolar bone ridge and is thus more prone to damage during surgical procedures. ^2^ ^,^ ^3^ However, the typical location of the mental foramen is reported to be either between the apices of the first and second premolars or below the apex of the second premolar. ^4^ This position of the mental foramen demonstrates anatomical variations, which can be found as far anterior as the canine ^5^ and posterior as the first molar. ^6^

The mental foramen is an important anatomical structure representing the termination of the mandibular canal. ^7^ Its accurate identification depends on knowledge of its location; this is strategically important for diagnostic and clinical procedures. ^8^ As a result, it appears that no single and consistent pattern of mental foramen placement exists across diverse groups. Hence, in clinical dental treatment, a detailed assessment of the mental location typical of each group is extremely useful. The shape, size, and position of the mental foramen must be evaluated, especially when considering various dental treatments performed on the mandible.

Currently, high-resolution 3D cone-beam computed tomography (CBCT) is the most promising and accurate technique available for quantitatively determining the location of mental foramen and the presence of anterior loops. ^9^

The high image quality of bone tissue and anatomical structure features provided by CBCT analysis reduces the risk of injury in the lower alveolar vascular nerve bundle. ^10^

Many authors and researchers in their retrospective studies assessed the details of the mental foramen (MF). ^11^ ^–^ ^14^ They have found that in very few cases, the exact position of the mental foramen can be observed in coronal, axial, sagittal, cross-sectional, and three-dimensional reconstructed images using CBCT.

It has been reported in the literature that race and ethnic variation are also visible in the mental foramen, accessory mental foramen, and anterior loop. ^12^ Considering this, there are variations expected in the Saudi population, yet few studies have reported variations. ^13^ ^,^ ^14^ Thus, this retrospective study was conducted to evaluate the shape, size, and location of mental foramen in dentulous and edentulous among the Saudi population using 3D CBCT. This study aimed to compare the positions and dimensions of MF openings between edentulous and dentate subjects matched by gender and nationality through CBCT.

## Methods

### Ethical approval

This study was approved by the Ethics Committee of the College of Dentistry (IRB number FPGRP/2020/508/318/319), Riyadh Elm University, Riyadh, Kingdom of Saudi Arabia. All patients sign a consent form before their appointments which states that radiographs and photographs are property of the college and may be used for teaching clinical demonstrations or scientific publications.

### Sample collection

In this retrospective study, to assess the relevant information of the mental foramen, CBCT scans taken between 2015 and 2020 from Riyadh Elm University (Database in Riyadh, Kingdom of Saudi Arabia) were analyzed. The required minimum sample (n=180) for the study was considered. One hundred and eighty scans fulfilling the inclusion criteria were selected. The following inclusion criteria were used:

▪ Only Saudi nationals
▪ Free from any systemic condition or systemic conditions which may have an impact on the result of the study
▪ Only permanent dentition
▪ Good quality CBCT with high volumetric data

The samples were then evenly sorted into two categories; group 1: dentate subjects; and group 2: edentulous subjects (unilateral or bilateral missing premolars). Variables such as gender and age were also considered in the group categories.

*CBCT data*: For standardization, all images were taken with a Sirona Galileos CBCT machine with exposure setting (85 kV, 28-35 mAs). CBCT images were exported in a digital imaging communication in medicine (DICOM) file format (.dcm). Images were accessed through on-demand 3D reconstruction imaging software (Version 8.0 205686 CyberMed inc, Seoul, Korea). Using CBCT panoramic reformatting images, tangential as well as cross-sectional view and nerve marking were done.

*Radiographic evaluation:* All the radiographic measurements were done by a single experienced CBCT reading examiner based at the university who is well trained in CBCT. To measure the reliability of the taken measurements, a second experienced examiner was asked to take the same readings following the described outlines for 20% of the final CBCT image scans. Inter-examiner reliability was assessed using Cronbach alpha before starting the assessment and considered acceptable (α>70%). The field of view (FOV) used in the present study was 5 × 5 cm.

The following measurements were taken using the modified method proposed by Zaman *et al.* (2016). ^15^

i. The vertical position of the mental foramen
  Three vertical lines were drawn on the longitudinal axis of the first premolar, second premolar, and mesial root of the first molar, respectively, to identify the horizontal position of the mental foramen. A horizontal line was drawn connecting the apices of the first and second premolars.
  The vertical position of MF was measured as follows:
  • Level A (LA) - Above the horizontal line.
  • Level B (LB) - At the horizontal line.
  • Level C (LC) - Below the horizontal line.
  • Mental foramen - Alveolar crest in mm.
  • Mental foramen - Apical point of lower cortical mandibular bone is also measured.
ii. The horizontal position of the mental foramen (when the premolars were missing, the approximate position of the premolars was considered)
  • Position 1 (P1) - Situated mesial to the long axis of the first premolar.
  • Position 2 (P2) - Situated in line with the long axis of the first premolar.
  • Position 3A (P3A) - Mesial 1/3 of position between the long axis line of the first and second premolar.
  • Position 3 (P3) - Middle 1/3 of position between the long axis line of the first and second premolar.
  • Position 3B (P3B) - Distal 1/3 of the position between the long axis line of the first and second premolar.
  • Position 4 (P4) - Situated in line with the long axis of the second premolar.
  • Position 5 (P5) - Between the long axis of the second premolar and the mesial root of the first molar.
  • Position 6 (P6) - Situated in line with the long axis of the mesial root of the first molar.
iii. Additional parameters
  • MFW (width) width of mental foramen in transverse section.
  • Emerging MF angle.

### Data analysis

Descriptive statistics for frequency, percentages, mean, and standard deviation were calculated for the various measures related to the mental foramen. For independent samples, a t-test was applied to compare mental foramen size, angle, and width between dentulous and edentulous jaws. The Mann-Whitney U test was applied to compare the mental foramen-related continuous variables on right and left sides, and between gender. All the data were analyzed by using SPSS version 25 (IBM-SPSS, Armonk, NY: USA, RRID: SCR_016479). A p-value of <0.05 was considered statistically significant.

## Results

**Figure 1.**
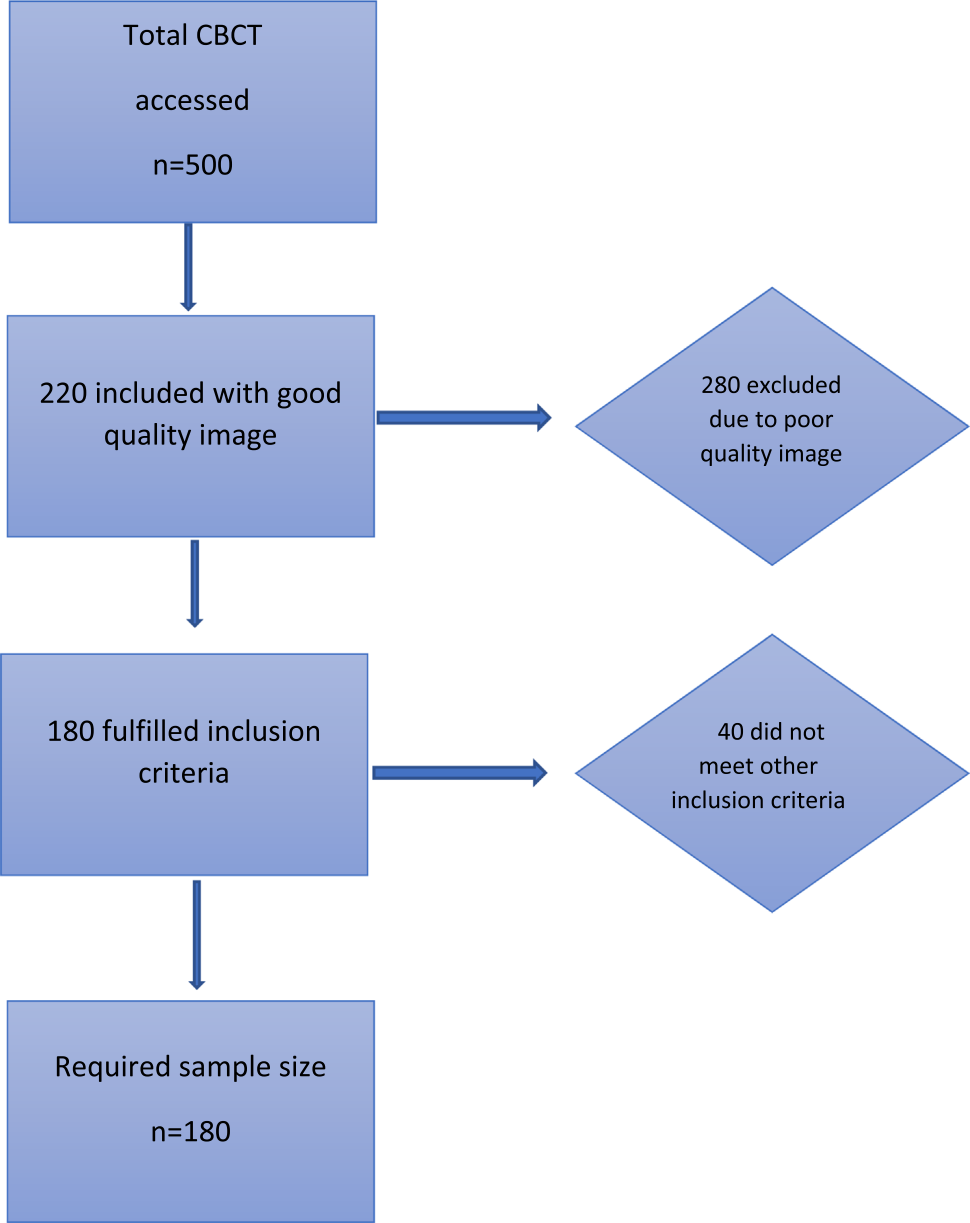
Participant selection. CBCT=cone-beam computed tomography.

From the total 500 scans, 280 were excluded as they were not good quality images and 40 as they did not fulfill other inclusion criteria such as Saudi nationals (n=22), free from systemic disease/condition (n=12), and permanent dentition (n=6). Hence, 180 were included in the analysis ( Figure 1).

The descriptive characteristics of the study variables are shown in Table 1. A total of 180 CBCT were analyzed, of which 96 were male and 84 were female. ^16^ Among 180 CBCT, it was ensured that there was equal distribution between dentate, edentulous, and sides (left and right) and vertical position among the CBCT is LC, horizontal position shown P4 having the highest percentage 67.8%.

**Table 1.** Characteristics of the study variables.

Variables		n	%
Gender	Male	96	53.3%
	Female	84	46.7%
	Total	180	100.0%
Group	Dentate	90	50.0%
	Edentulous	90	50.0%
	Total	180	100.0%
Side	Left side	180	50.0%
	Right side	180	50.0%
	Total	360	100.0%
Vertical Position	LC	180	100.0%
	Total	180	100.0%
Horizontal Position	P1	0	0.0%
	P2	4	1.1%
	P3A	2	0.6%
	P3	60	16.7%
	P3B	14	3.9%
	P4	244	67.8%
	P5	34	9.4%
	P6	2	0.6%
	Total	360	100.0%

• Level C (LC) - Below the horizontal line. • Position 1 (P1) - Situated mesial to the long axis of the first premolar. • Position 2 (P2) - Situated in line with the long axis of the first premolar. • Position 3A (P3A) - Mesial 1/3 of position between the long axis line of the first and second premolar. • Position 3 (P3) - Middle 1/3 of position between the long axis line of the first and second premolar. • Position 3B (P3B) - Distal 1/3 of position between the long axis line of the first and second premolar. • Position 4 (P4) - Situated in line with the long axis of the second premolar. • Position 5 (P5) - Between the long axis of the second premolar and mesial root of the first molar. • Position 6 (P6) - Situated in line with the long axis of the mesial root of the first molar.

59 males and 63 females showed a P4 horizontal position. A statistically significant association was found between the horizontal position of the mental foramen and gender (p<0.05) ( Table 2). A statistically significant association was also found between the horizontal position of the mental foramen and dentate status (p<0.05) ( Table 3). On the other hand, no statistically significant association was found between the horizontal position of the mental foramen and sides (p>0.05) ( Table 4).

**Table 2.** The horizontal position of the mental foramen between gender.

		P2	P3A	P3	P3B	P4	P5	P6	Total	p-value
Male	n	2	0	15	7	59	12	1	96	0.016
	%	1.1	0.0	8.3	3.9	32.8	6.7	0.6	53.3	
Female	n	0	1	15	0	63	5	0	84	
	%	0.0	0.6	8.3	0.0	35.0	2.8	0.0	46.7	
Total	n	2	1	30	7	122	17	1	180	
	%	1.1	0.6	16.7	3.9	67.8	9.4	0.6	100	

**Table 3.** The horizontal position of the mental foramen between dentate and edentulous jaws.

		P2	P3A	P3	P3B	P4	P5	P6	Total	p-value
Dentate	N	2	1	24	7	43	13	0	90	<0.001
	%	1.1	0.6	13.3	3.9	23.9	7.2	0.0	50.0	
Edentulous	N	0	0	6	0	79	4	1	90	
	%	0.0	0.0	3.3	0.0	43.9	2.2	0.6	50.0	
Total	n	2	1	30	7	122	17	1	180	
	%	1.1%	0.6	16.7	3.9	67.8	9.4	0.6	100.0	

**Table 4.** The horizontal position of the mental foramen between the right and left sides.

		P2	P3A	P3	P3B	P4	P5	P6	Total	p-value
Left side	n	4	2	22	8	126	16	2	180	0.378
	%	1.1	0.6	6.1	2.2	35.0	4.4	0.6	50.0	
Right side	n	0	0	38	6	118	18	0	180	
	%	0.0	0.0	10.6	1.7	32.8	5.0	0.0	50.0	
Total	n	4	2	60	14	244	34	2	360	
	%	1.1%	0.6	16.7	3.9	67.8	9.4	0.6	100.0	

The mean and standard deviation (SD) mental foramen angle was highest on the left side in the 50-59-year-old group in the edentulous group (53.95±13.97) and least on the right side in the 16-19-year-old group in the dentulous group (38.00±8.26) ( Table 5). The mean distance of the mental foramen to the alveolar crest (MF_MSB) was statistically significantly higher (t=6.235, p<0.001) in males (14.52±3.29 mm) than in females (11.22±3.80 mm). In addition, the mean distance of the mental foramen to the mandibular inferior border of the mandible (MF_MIB) was statistically significantly higher (t=2.875, p=0.005) in males (11.59±2.10 mm) than in females (10.25±3.95 mm). Moreover, the mean width of the mental foramen in the transverse section (MFW) was statistically significantly higher (t=3.622, p<0.001) in males (3.43±0.82 mm) than in females (2.99±0.83 mm). However, the mean M angle between males (44.21±9.06) and females (46.11±12.03) was not found to be statistically significant (t=-1.205, p=0.239). The mean MF_MSB, MF_MIB, and MFW were statistically significantly higher in males (p<0.05) ( Table 6).

**Table 5.** The right and left mental foramen angle according to age.

Age (Years)	Side	n	Mean	SD
16-19	Left	6	43.90	8.66
	Right	6	38.00	8.26
20-29	Left	72	42.30	9.00
	Right	72	42.29	8.83
30-39	Left	30	43.61	12.30
	Right	30	41.09	8.16
40-49	Left	10	50.48	11.44
	Right	10	46.76	10.26
50-59	Left	26	53.95	13.97
	Right	26	52.02	11.43
60-69	Left	18	46.46	11.32
	Right	18	44.46	10.49
70-79	Left	18	50.78	10.57
	Right	18	46.24	6.20

**Table 6.** Comparison of study variables between gender.

		n	Mean	SD	SEM	t	p-value
MF_MSB	Male	96	14.52	3.29	0.34	6.235	<0.001
	Female	84	11.22	3.80	0.41		
MF_MIB	Male	96	11.59	2.10	0.21	2.875	0.005
	Female	84	10.25	3.95	0.43		
MFW	Male	96	3.43	0.82	0.08	3.622	<0.001
	Female	84	2.99	0.83	0.09		
M angle	Male	96	44.21	9.06	0.93	-1.205	0.239
	Female	84	46.11	12.03	1.31		

MF-MSB (mental foramen to alveolar crest); MF-MIB (mental foramen to mandibular inferior border of mandible); MFW (width of mental foramen in transverse section); MF angle (mental foramen angle).

The mean distance of MF_MSB was found to be statistically significant (t=8.763, p<0.001) higher in dentate (15.11±2.72 mm) than in edentulous (10.85±3.73 mm). On the other hand, the mean distance of MF_MIB between dentate (11.36±2.16 mm) and edentulous (10.57±3.90 mm) was not found to be statistically significant (t=1.688, p=0.093). The mean MFW between dentate (3.46±0.74 mm) and edentulous (2.99±0.89 mm) was found to be statistically significantly higher in dentate (t=3.815, p<0.001). Furthermore, the mean M angle between dentate (41.92±8.33) and edentulous (48.27±11.61) was found to be statistically significant (t=-4.217, p<0.001) ( Table 7). There was no statistically significant difference in the mean of all study variables by sides (p>0.05) ( Table 8).

**Table 7.** Comparison of study variables between dental status.

		n	Mean	SD	SEM	t	p-value
MF_MSB	Dentate	90	15.11	2.72	0.29	8.763	<0.001
	Edentulous	90	10.85	3.73	0.39		
MF_MIB	Dentate	90	11.36	2.16	0.23	1.688	0.093
	Edentulous	90	10.57	3.90	0.41		
MFW	Dentate	90	3.46	0.74	0.08	3.815	<0.001
	Edentulous	90	2.99	0.89	0.09		
MF angle	Dentate	90	41.92	8.33	0.88	-4.217	<0.001
	Edentulous	90	48.27	11.61	1.22		

MF-MSB (mental foramen to alveolar crest); MF-MIB (mental foramen to mandibular inferior border of mandible); MFW (width of mental foramen in transverse section); MF angle (mental foramen angle).

**Table 8.** Comparison of study variables between sides.

		n	Mean	SD	SEM	t	p-value
MF_MSB	Left	180	13.02	4.10	13.02	0.123	0.902
	Right	180	12.95	3.70	12.95		
MF_MIB	Left	180	10.61	2.15	0.23	-1.506	0.134
	Right	180	11.32	3.91	0.41		
MFW	Left	180	3.24	0.90	0.09	0.317	0.752
	Right	180	3.20	0.80	0.08		
M angle	Left	180	45.97	11.42	1.20	1.119	0.264
	Right	180	44.21	9.62	1.01		

MF-MSB (mental foramen to alveolar crest); MF-MIB (mental foramen to mandibular inferior border of mandible); MFW (width of mental foramen in transverse section); MF angle (mental foramen angle).

## Discussion

A good surgeon always plans the surgical procedure using required diagnostic aids and gives attention to each small anatomical structure in the surgical field, including anatomical variation (if any) to avoid surgical complications. The mandible, with its associated anatomical structures in the posterior region, always requires additional attention to avoid injury to the neurovascular bundles and any complications related to them. The mental foramen and associated alterations should have been given attention before surgery. Anatomically, mental foramen shows a lot of variation with the number of foramina and accessory foramina and the possibility of the presence of an anterior loop of the mental nerve. ^4^

In the present study, we have utilized CBCT to assess the position of the mental foramen. Many previous studies evaluating the mental foramen and its associated structure were done in cadavers, and human studies using panoramic radiographs, ultrasonography, and CBCT. ^8^ ^–^ ^14^ Panoramic radiographs have their disadvantages with a lack of clarity, thus smaller mental foramen may go unnoticed. CBCT was the radiographic method of choice in the present study because it reveals anatomical structures without superimpositions and deformation, which are observed in traditional imaging techniques such as panoramic image analysis and at low levels of radiation. ^17^

Although several studies are available regarding the mental foramen and its associated anatomical variation which are carried out within the Saudi population. All studies done only among dentate subjects. This is the first study exclusively comparing the dentate and edentulous. Furthermore, initial studies were done using panoramic views. ^11^ ^–^ ^14^ However, there are drawbacks of using a panoramic view as previously outlined and the result may vary when they are compared with that of the CBCT. ^5^

The prevalence of horizontal position of mental foramen in the present study predominated by P4 (35% in males and 32.8% in females), followed by P3 (8.3% each in males and females). P4 appears to be the most common position, or more prevalent position, seen in almost all the studies which are done among Saudi, Jordanian, and Egyptian populations. ^13^ ^,^ ^18^ However, variation in the position was seen in studies among the Indian (P5), European (P3), and Iranian populations (P3). In the present study, the prevalence was similar to the study of Alam *et al.* (2018). ^13^ However, many other studies have shown higher prevalence. The similarity and difference in the position of mental foramen are related to race and potentially due to change in the sample size of the age group. Hence, the percentage of prevalence seen in this study may be different than the previous studies.

In the present study, the variation in the mental foramen compared between males and females was found to be statistically significant. The horizontal position of the mental foramen and gender showed a statistical significance difference, especially at the P3B, P5, and P4 positions. There was a statistically significant difference seen in MF_MSB and MFW. However, there is no significant difference in MF_MIB and MF angles. Differences in the position of mental foramen across gender and racial groups have also been reported in the literature. Present study results are similar to some parameters which were presented in Alam *et al.* (2018). ^13^ In their study, the P4 position is more prevalent in males which is similar to our study, but they have found P3 to be more prevalent in females. However, in our study, we have found P4 more commonly prevalent, followed by P3. There was a also statistical difference in P3B and P5 prevalence among males and females. A study done in the Indian population has shown slightly different results, where they have found P4 followed by P3 being common in males on the right side of the mandible and P4 for the females on the left side of the mandible. ^19^ Variation in the result may be due to the difference in the race of the population studied.

Li *et al.* (2018) and Al-Mahalawy *et al.* (2017) found almost similar results to the present study except for the finding of the prevalence among gender. ^12^ ^,^ ^20^ In a study of the Polish population, the average values of horizontal and vertical diameter for males were significantly higher on the right side than in the female subgroup. Whereas, on the left side the average value of only the vertical diameter was significantly higher in males compared to women. ^21^ Thus, various studies have pointed out the difference among gender as found in the present study. Variability in the prevalence among males and females was not consistent, with few studies mentioning no difference and few other studies mentioning differences between the gender. The difference in the result could be related to the age group of the sample, sample size, and racial predilection.

The present study is unique compared to many other studies because we have taken both dentate and edentulous patients. It is known that there is variation in the location of the mental foramen as the age advances due to resorption of the crestal bone or change in the position of the mandible due to differences in the growth of the mandible. In the present study, although we have found the prevalent position is P4 in both the dentate and edentulous groups, this result of the study is to be read cautiously due to the difference in the sample size chosen.

With regards to the age effect on the morphology of the foramina, studies have shown different results, although many studies did not specify the group as dentate or edentulous, the age group variation has been shown. ^11^ ^–^ ^14^ Differences in the division of the age group could lead to different conclusions ^22^ ^,^ ^23^ on variation in the mental foramen location, number of mental foramina, and anterior loop of the mental nerve.

One of the studies revealed that age-related differences in the accessory mental foramen size varied between pediatric and adult populations due to the growth process of the mandible ^24^; variation in the edentulous and dentate subject across age variation is an important consideration during implant placement. In the present study, there was no statistical significance in the MF position between the left and right sides in the horizontal position. However, we have seen a difference in the results for MF_ MSB, M angle, and MFW. The present study results are similar to the studies reported among the Jordanian, Saudi, and Iranian populations, ^13^ ^,^ ^25^ but a few other studies reported the prevalence of asymmetric mental foramen in Saudi, Egyptian, and Jordanian populations too. ^5^ ^,^ ^26^ The variation in the position of mental foramen in different studies may be due to racial variations and factors which may have influenced the growth of either side differently. ^13^

The variation in the distance between the mental foramen to the alveolar crest, relation to the lower border of the mandible, and corresponding changes in the width of mental foramen in the transverse section is commonly increased by age. The mean distance from the upper border of the mental foramen to the alveolar crest in our study is 15.11 mm in dentate and 10.85 mm in edentulous, which is almost in the range of the previous study done the Saudi population ^12^ where the range was 9.1–19.2 mm (Mean: 14.3 mm). Similar reports have been published by Haktanir *et al.* (2010), which showed a mean distance of 14.2 mm (Range: 10.7–29.8 mm), ^27^ where the lower range is close to our study, but there was a much larger difference observed in the upper range.

Changes in the vertical distance seen in this study, especially in the upper range, could be due to the resorption of the alveolar crest, age of the patient, growth pattern changes in the mandible as age advances, and genetic changes. ^12^ To overcome the shadow of the alveolar crest resorption having an impact on this parameter, one author suggested the use of cementoenamel junction (CEJ) of adjacent teeth as a guide. ^28^

The mean width of the mental foramen in the transverse section (MFW) was statistically significantly higher (t=3.622, p<0.001) in males (3.43±0.82 mm) than females (2.99±0.83 mm) Lopes *et al*. ( 2016) ^29^ in their study found similar results with MFW or size is increased in the males, they have also pointed out that, the edentulism only reduced dimension of mental foramen opening. 

Similar to the results of the relation between the mental foramen and alveolar crest, many studies established and presented the distance between the mental foramen and the inferior border of the mandible. In our study, we have seen a mean distance of 10.57 mm in the edentulous and 11.36 mm in the dentate subjects. These study results are again similar to the study reports of Al-Mahalawy *et al.* (2017), where they reported the mean distance between the inferior margin of mental foramen and lower mandibular border was 13.8 mm (Range: 8.7–16.6 mm). ^12^ Similarly in the reports by Von Arx *et al.* (2013) and Kalender *et al.* (2012), the average distance was found to be 13.2 mm and 12.4 mm, respectively. ^30^ ^,^ ^31^

There are some limitations of the study. The sample size of the study though drawn after considering the value of previous studies, increasing the sample size may be helpful to draw a better conclusion. Further, we have considered the dentate and edentulous age group to be a limited range, since the anatomy of mental foramen may vary from age to age. A further age range inclusion may broaden the result with a better conclusion. Considering the similarity in some of the parameters in the same ethnic population, differences across ethnic populations, and changes in the age group, gender, and state of dentate and edentulous, a recent systematic review on the anterior loop and mental foramen rightly pointed out that there is no fixed parameter to be relied on for the presence and distribution of anterior loop. ^8^ Though it appears that in a given ethnic population and age group some parameters appear to be similar, it is highly recommended not to rely on any average values available for the anterior loop. The clinician is advised to use imaging modalities available in every case wherever surgical procedure is to be performed near the mental foramen region for identification and accurate measurements of the anterior loop length to avoid any injury. ^8^ If clinicians want to avoid complications and extend comfort to the patient, it is prudent to be cautious during the operation. Clinicians should be on the lookout for unanticipated deviations, especially when doing dental operations that entail periosteal detachment and implant placement in the mental area.

## Conclusions

Within the limitation of the study, it can be concluded that the horizontal position of the mental foramen is predominated by P4 followed by P3 in this sample of the Saudi population. Variation in the position of the mental foramen among dentate and edentulous subjects was seen. Considering the variations in the age group, gender, and between dentate and edentulous subjects, the clinician needs to be careful about the position of the mental foramen and the use of CBCT as a diagnostic aid before the surgical procedure needs to be considered.

## Data Availability

Access to the underlying data is only available via the database at Riyadh Elm University. To access the database, researchers must contact the College of Dentistry at Riyadh Elm University to request access. Researchers must be the faculty of the said university to apply for data access to the authorities. A summary of the data used in this study is available in the underlying data statement. Harvard Dataverse. Evaluation of Shape, Size and Location of a Mental Foramen in dentulous and edentulous among Saudi population Using 3D Cone-Beam Computed Tomography. https://doi.org/10.6084/m9.figshare.21275934.v1. ^16^ This project contains the following underlying data:
• GR 1 dentate left.tab. (underlying data for group 1 dentate – left side) • GR 1 R DENTATE.tab. (underlying data for group 1 dentate – right side) • GR 2 LEFT.tab. (underlying data for group 2 endentulous – left side) • GROUP 2 R.tab. (underlying data for group 2 endentulous – right side) Data are available under the terms of the Creative Commons Zero “No rights reserved” data waiver (CC0 1.0 Public domain dedication).
