# Supplementary material for: Evaluation of shape, size, and location of mental foramen in dentulous and edentulous among Saudi population using 3D cone-beam computed tomography
Source: F1000Res. 2022 Nov 30;11:916. Originally published 2022 Aug 9. [Version 2] doi: 10.12688/f1000research.74434.2 (PMC12754353; doi:10.12688/f1000research.74434.2)
Supplement: Peer review history

# Reviewer report: Reviewer response for version 2

**DOI:** 10.5256/f1000research.139524.r439584

**Published:** 2025-12-30

**Authors:** Ashwin Shetty Yermal

**Affiliations:** 1 Imam Abdulrahman Bin Faisal University, Dammam, Eastern Province, Saudi Arabia

Interesting study on the evaluation of shape, size, and location of mental foramen in dentulous and edentulous individuals among the Saudi population using 3D CBCT, with the following observation:

 1. Need to remove repetitive information in the introduction.

 2. Need to elaborate on the justification for the study. 

 3. Need to elaborate on the sample size and power calculation.

 4. Need to mention the criteria for excluding specific systemic conditions in the exclusion criteria.

 5. Discussion is too long

 6. Conclusion needs to be more specific.

 7. Need to correct grammatical errors.

Is the work clearly and accurately presented and does it cite the current literature?

Yes

If applicable, is the statistical analysis and its interpretation appropriate?

Yes

Are all the source data underlying the results available to ensure full reproducibility?

Yes

Is the study design appropriate and is the work technically sound?

Yes

Are the conclusions drawn adequately supported by the results?

Yes

Are sufficient details of methods and analysis provided to allow replication by others?

Yes

Reviewer Expertise:

Dental anomalies, dental public health.

I confirm that I have read this submission and believe that I have an appropriate level of expertise to confirm that it is of an acceptable scientific standard.

# Reviewer report: Reviewer response for version 2

**DOI:** 10.5256/f1000research.139524.r426775

**Published:** 2025-11-12

**Authors:** Junaid Ahmed

**Affiliations:** 1 Department of Oral Medicine & Radiology, Manipal College of Dental Sciences, Mangalore, Karnataka, India

1.In Abstract section, under heading of 'Results', in the following sentence- 'mental foramen to the mandibular inferior border of the mandible (MF_MSB)',is the abbreviation of inferior border of mandible-'MSB'?

 2.The authors need to elaborate on the need for the study more clearly with an emphasis on the research gaps especially with regards to its clinical implications.

 3.The authors need to clearly state the methodology employed for sample size estimation and quote the reference article/study if any.

 4.In the Inclusion criteria, the authors need to specify what do they exactly mean by 'good quality CBCT'.

 5.In the Inclusion criteria, the authors need to be more specific about the systemic conditions they have excluded. Infact, the criteria of the specific systemic conditions being excluded should be included in the exclusion criteria which unfortunately has not been mentioned separately.

 6.In the Results section, how did the authors include 180 of 500 scans when they excluded 360 of them?

 7.In the conclusion section, the authors mention-'...be careful about the position of the mental foramen and the use of CBCT', which implies that the clinician should be careful in using CBCT? ,is this what the authors want to convey?

 8.Throughout the manuscript, there are quite a few grammatical errors and sentences that need to be rephrased for a better understanding.

Is the work clearly and accurately presented and does it cite the current literature?

Partly

If applicable, is the statistical analysis and its interpretation appropriate?

Yes

Are all the source data underlying the results available to ensure full reproducibility?

Partly

Is the study design appropriate and is the work technically sound?

Yes

Are the conclusions drawn adequately supported by the results?

Yes

Are sufficient details of methods and analysis provided to allow replication by others?

Yes

Reviewer Expertise:

Digital Radiology,Oral Cancer,Forensic Odontology

I confirm that I have read this submission and believe that I have an appropriate level of expertise to confirm that it is of an acceptable scientific standard, however I have significant reservations, as outlined above.

# Reviewer report: Reviewer response for version 2

**DOI:** 10.5256/f1000research.139524.r417388

**Published:** 2025-10-03

**Authors:** Svetlana Antic

**Affiliations:** 1 Radiology, University of Belgrade Faculty of Dental Medicine (Ringgold ID: 119082), Belgrade, Serbia, Serbia

This is an interesting study aiming to compare the shape, size and positions of mental foramen openings between dentate and edentelous individuals.

  Title: 

 The proper pairing is **dentate and edentulous**, not "dentelous."

 Introduction: The limited number of existing studies on this topic is not a sufficient justification for conducting the present research. It is essential to highlight the unknown aspects of the subject and direct part of the investigation toward addressing them, which will enhance the study’s novelty and scientific contribution.

 Methods:

 1) How did you calculate the required sample size? Which tool/software did you use and which data from the literature served as the basis for your calculation?

 2) Please provide one CBCT image illustrating the measurement method, with reference lines clearly marked and with presented examples of the determined positions

 Discussion

 You stated that the present study is unique compared to many other studies due to comparison of dentate and edentulous patients. Explain why this issue is important  in relation to the most common surgical procedures in dentate and edentulous patients.

Is the work clearly and accurately presented and does it cite the current literature?

Partly

If applicable, is the statistical analysis and its interpretation appropriate?

I cannot comment. A qualified statistician is required.

Are all the source data underlying the results available to ensure full reproducibility?

Yes

Is the study design appropriate and is the work technically sound?

Partly

Are the conclusions drawn adequately supported by the results?

Yes

Are sufficient details of methods and analysis provided to allow replication by others?

Yes

Reviewer Expertise:

Oral radiology, Maxillo-facial radiology, Head & neck radiology

I confirm that I have read this submission and believe that I have an appropriate level of expertise to confirm that it is of an acceptable scientific standard, however I have significant reservations, as outlined above.

# Reviewer report: Reviewer response for version 1

**DOI:** 10.5256/f1000research.78190.r147197

**Published:** 2022-09-13

**Authors:** Astha Chaudhry

**Affiliations:** 1 Department of Oral Medicine and Radiology, Faculty of Dental Sciences, Shree Guru Gobind Singh Tricentenary (SGT) University, Gurugram, Haryana, India

Thank you for giving me the opportunity to review the manuscript. The following are the observations:

1. The title of manuscript is " Evaluation of shape, size, and location of mental foramen in dentulous and edentulous among Saudi population using 3D cone-beam computed tomography". However, I do not find any mention of shape of mental foramen or its alterations in the introduction or methodology. The location of foramen is extensively described and for size only the horizontal width(MFW) has been considered.
2. The Introduction is haphazard with lots of irrelevant and redundant and repetitive information. 
  Some factual errors are also present like it is mentioned "Mental foramen is typically located on **lingual **side". It is located buccally. And "the anterior canal and submandibular fossa **convexity**" , the submandibular gland fossa will show concavity etc.
3. The authors have tried to add too much information related to mental foramen in the introduction which is not relevant to the objectives of the study.
4. It is mentioned that "the high image quality of bone tissue and anatomical structure features provided by CBCT analysis reduces the risk of lesions in the lower alveolar vascular nerve bundle". This is difficult to comprehend, as the good image quality of CBCT cannot reduce the risk of lesions in nerve. Lots of rephrasing and paraphrasing is required.
5. Regarding the methodology, few basic things are not clear:
   
  - What type of edentulous patients have been included in the study, is it completely edentulous or partially edentulous and if partially edentulous, does it involve premolars??
  - If its completely edentulous or partially edentulous involving any one or both the premolars, how did one assess the horizontal position P1 to P6 as the reference premolars/teeth are absent and if the edentulism does not involve the premolars, how does it affect the mental foramen? Since, the authors have claimed at multiple points in the manuscript that the USP of the study is comparison between dentate and edentulous populations, it is all the more important to clarify these in the methodology.
  - What FOV scans were included in the study? Must be mentioned in the inclusion criteria.
  - As the authors claimed that they have evaluated both the sides in one patient scan, means in total they have evaluated 360 mental foramina. There can be variations in the size, shape, and position of foramen on both the sides, also the presence or absence of teeth. Thus, how can one assess the association of the horizontal position of foramen with gender (table 2), the horizontal position of foramen between dentate and edentulous (table 3), and parameters in Table 6 and table 7. Was the average of the right and left side taken into consideration? It has not been clearly explained in the methodology. It is difficult to take out any relevant inference through these tables. I believe the calculations should be made for 360 foramina evaluated rather than 180 cases as there can be individual variations on both the sides.
  - The authors have calculated mental foramen angle. It is not explained how? 
    It would have been better if some images, pictures are provided how the measurements will be taken. Also highlight what is the significance of calculating this angle.
  - The measurements mentioned in the methodology mention the distance between mental foramen and alveolar crest in vertical position which is same as MF-MIB and similarly MF MSB and distance between foramen and lower cortical mandibular bone.
  - Why is only the horizontal width of the foramen considered and not vertical height? The authors have mentioned significant change in MFW in the edentulous cases, what is the reason? nothing has been explained clearly.  
6. The Discussion needs to be more focused.
7. The conclusion is not concrete and not specific. The vertical position variations according to age and edentulism are a proven fact. The reasons for the change in horizontal positions and its association with edentulism is not clear.
  The size variation is mentioned in the conclusion in the abstract but not adequately discussed in the discussion section.
  The shape variation has not been discussed at all, hence it should be removed from the title and aim and objectives.

Is the work clearly and accurately presented and does it cite the current literature?

Partly

If applicable, is the statistical analysis and its interpretation appropriate?

Partly

Are all the source data underlying the results available to ensure full reproducibility?

No

Is the study design appropriate and is the work technically sound?

No

Are the conclusions drawn adequately supported by the results?

No

Are sufficient details of methods and analysis provided to allow replication by others?

No

Reviewer Expertise:

Oral Radiology, CBCT, Oral Medicine

I confirm that I have read this submission and believe that I have an appropriate level of expertise to state that I do not consider it to be of an acceptable scientific standard, for reasons outlined above.

# Response

**Published:** 2022-10-05

**Authors:** Salim S

1. The title of manuscript is " Evaluation of shape, size, and location of mental foramen in dentulous and edentulous among Saudi population using 3D cone-beam computed tomography". However, I do not find any mention of shape of mental foramen or its alterations in the introduction or methodology. The location of foramen is extensively described and for size only the horizontal width(MFW) has been considered.
**Response:** word shape is removed, irrelevant discussion on other factors removed and we intended to consider only horizontal width, which was not discussed in the Saudi population till now properly.
2. The Introduction is haphazard with lots of irrelevant and redundant and repetitive information. 
**Response: **all irrelevant materials have been removed.
  Some factual errors are also present like it is mentioned "Mental foramen is typically located on **lingual **side". It is located buccally. And "the anterior canal and submandibular fossa **convexity**" , the submandibular gland fossa will show concavity etc.
**Response: **corrected. the second sentence containing this information is removed.
3. The authors have tried to add too much information related to mental foramen in the introduction which is not relevant to the objectives of the study.
**Response: **taken out, only required aspects are there.
4. It is mentioned that "the high image quality of bone tissue and anatomical structure features provided by CBCT analysis reduces the risk of lesions in the lower alveolar vascular nerve bundle". This is difficult to comprehend, as the good image quality of CBCT cannot reduce the risk of lesions in nerve. Lots of rephrasing and paraphrasing is required.
**Response:** removed the word lesion and written as injury.
5. Regarding the methodology, few basic things are not clear:
   
  - What type of edentulous patients have been included in the study, is it completely edentulous or partially edentulous and if partially edentulous, does it involve premolars??
**Response:** dentate subjects; and group 2: edentulous subjects (unilateral or bilateral missing premolars).
  - If its completely edentulous or partially edentulous involving any one or both the premolars, how did one assess the horizontal position P1 to P6 as the reference premolars/teeth are absent and if the edentulism does not involve the premolars, how does it affect the mental foramen? Since, the authors have claimed at multiple points in the manuscript that the USP of the study is comparison between dentate and edentulous populations, it is all the more important to clarify these in the methodology.-not completely edentulous, partially edentulous. Position P1 TO P6  as taken as approximate in case of absence of premolars
**Response:** edentulous area involves premolars only.
  - What FOV scans were included in the study? Must be mentioned in the inclusion criteria.
**Response:** mentioned   
  - As the authors claimed that they have evaluated both the sides in one patient scan, means in total they have evaluated 360 mental foramina. There can be variations in the size, shape, and position of foramen on both the sides, also the presence or absence of teeth. Thus, how can one assess the association of the horizontal position of foramen with gender (table 2), the horizontal position of foramen between dentate and edentulous (table 3), and parameters in Table 6 and table 7. Was the average of the right and left side taken into consideration? It has not been clearly explained in the methodology. It is difficult to take out any relevant inference through these tables. I believe the calculations should be made for 360 foramina evaluated rather than 180 cases as there can be individual variations on both the sides.
**Response:** as one can see - a total of 360 scans were included and analyzed (180 numbers of CBCT)
  - The authors have calculated mental foramen angle. It is not explained how? 
**Response:** as per the previous author's reference (Zaman et al. 2016)
    It would have been better if some images, pictures are provided how the measurements will be taken. Also highlight what is the significance of calculating this angle.
**Response:** the significance of calculating the angle is for a better location.
  - The measurements mentioned in the methodology mention the distance between mental foramen and alveolar crest in vertical position which is same as MF-MIB and similarly MF MSB and distance between foramen and lower cortical mandibular bone.
**Response:** since these readings are repeated this is eliminated from the methods.
  - Why is only the horizontal width of the foramen considered and not vertical height? The authors have mentioned significant change in MFW in the edentulous cases, what is the reason? nothing has been explained clearly.
**Response:** we intended to locate the mental foramen through horizontal width since the vertical position variations according to age and edentulism are a proven fact.
6. The Discussion needs to be more focused.
7. The conclusion is not concrete and not specific. The vertical position variations according to age and edentulism are a proven fact. The reasons for the change in horizontal positions and its association with edentulism is not clear.
**Response:** conclusion changed.
  The size variation is mentioned in the conclusion in the abstract but not adequately discussed in the discussion section.
**Response:** included.
  The shape variation has not been discussed at all, hence it should be removed from the title and aim and objectives.
**Response:** removed.

# Reviewer report: Reviewer response for version 1

**DOI:** 10.5256/f1000research.78190.r147200

**Published:** 2022-09-05

**Authors:** Mohammed Mashyakhy

**Affiliations:** 1 Department of Restorative Dental Sciences, College of Dentistry, Jazan University, Jazan, Saudi Arabia

- The introduction is too long and not related fully to the subject, its very wide, need to be concise on the topic
- Material and methods were not clear and confusing
- The results are ok 
- Discussion is not very clear and direct
- There are some studies from Saudi population using CBCT not mentioned
- I recommend the authors to focus on the aim and be concise especially in the material and methods

Is the work clearly and accurately presented and does it cite the current literature?

Partly

If applicable, is the statistical analysis and its interpretation appropriate?

I cannot comment. A qualified statistician is required.

Are all the source data underlying the results available to ensure full reproducibility?

Partly

Is the study design appropriate and is the work technically sound?

Partly

Are the conclusions drawn adequately supported by the results?

Partly

Are sufficient details of methods and analysis provided to allow replication by others?

Partly

Reviewer Expertise:

Dental anatomy, endodontics

I confirm that I have read this submission and believe that I have an appropriate level of expertise to state that I do not consider it to be of an acceptable scientific standard, for reasons outlined above.

# Response

**Published:** 2022-10-05

**Authors:** Salim S

- The introduction is too long and not related fully to the subject, its very wide, need to be concise on the topic
**Response:** deleted most of the irrelevant aspect and presented only required (reduced to more than 50%)
- Material and methods were not clear and confusing
**Response:** many of the irrelevant aspects are removed and made it easily understandable.
- The results are ok 
- Discussion is not very clear and direct
**Response:** made it almost clear and to the point
- There are some studies from Saudi population using CBCT not mentioned
**Response:** thanks for the observation, we did not include them since they were assessed in OPG
- I recommend the authors to focus on the aim and be concise especially in the material and methods
**Response:** removed unnecessary aspects and only relevant information in the materials and methods kept.
